# Multidimensional cognitive reserve and cognitive outcomes in glioblastoma: a pre- and postoperative analysis

**DOI:** 10.1007/s11060-026-05475-w

**Published:** 2026-02-21

**Authors:** Sophie Rauch, Yizhou Wan, Ajay Halai, Tom Manly, Haiyan Zheng, Roxanne Mayrand, Rohitashwa Sinha, Alexis Joannides, Richard Mair, Robert Morris, Thomas Santarius, Matthew Lambon-Ralph, Stephen J. Price

**Affiliations:** 1 Cambridge Brain Tumour Imaging Lab, Division of Neurosurgery, Department of Clinical Neurosciences, University of Cambridge, Cambridge, UK; 2 Division of Neurosurgery, Department of Clinical Neurosciences, University of Cambridge, Cambridge, UK; 3 MRC Cognition Brain Sciences Unit, Cambridge, UK; 4 Department of Mathematical Sciences, University of Bath, Bath, BA2 7AY UK; 5 Queens’ College, Cambridge, UK

**Keywords:** Cognitive reserve, Glioblastoma, Cognition, Neuropsychological assessment

## Abstract

**Purpose:**

Glioblastoma (GBM) is the most aggressive primary brain tumour in adults. One factor which is widely considered to have protective effects for cognition in age-related decline is cognitive reserve. However, little research has been done into the relationship between cognitive reserve and cognitive function in GBM. We investigated whether a multidimensional construct of cognitive reserve predicts cognitive outcomes before and after surgery.

**Method:**

43 adult patients with GBM (mean age = 62) took the Cognitive Reserve Index Questionnaire and participated in the OCS-BRIDGE cognitive screen at preoperatively, at 72 h and 6–8 weeks postoperatively. Linear regressions and mediation analyses assessed the relationship between cognitive reserve proxies (education, work and leisure) and cognitive performance across domains.

**Results:**

Higher overall cognitive reserve significantly predicted better preoperative performance in global deficit, executive function, memory and recognition memory. Mediation analysis revealed that education had a direct effect on executive function, independent of lifestyle activities. Postoperatively, most protective effects of cognitive reserve diminished. However, recognition memory at 72 h remained significantly associated with cognitive reserve, specifically work and leisure activities, as revealed by the mediation analysis.

**Conclusion:**

Cognitive reserve protects against tumour-related cognitive deficits, and in executive function, this is primarily driven by education. In contrast, “active” reserve components, such as work and leisure, may support recognition memory during the acute postoperative phase. These findings highlight the distinct clinical utility of multidimensional reserve assessment for preoperative risk stratification and potential prehabilitation.

## Introduction

Glioblastoma (GBM) (WHO grade 4) is the most aggressive and common primary brain cancer in adults [1], with a median survival of 15 months, and a typical onset around age 64 years [2–4]. Given the poor prognosis and substantial treatment burden associated with GBM, cognitive impairments are a major concern, affecting quality of life (QOL), daily functioning, and independence [5–7].

Similar to dementia and neurodegenerative conditions, GBM often results in significant cognitive decline [8]. In dementia and neurodegenerative conditions, cognitive reserve has emerged as a protective factor [9]. Cognitive reserve is distinct from the passive and fixed concept of brain reserve, which reflects structural characteristics of an individual’s neurobiology, such as the number of neurons or synapses [10]. Instead, cognitive reserve refers to the brain’s ability to cope with age-related changes or damage by flexibly recruiting neural networks [10]. This allows an individual to maintain cognitive function through more efficient use of their existing brain pathways or by engaging alternative, compensatory neural strategies. Since cognitive reserve cannot be measured directly, it is typically estimated using proxies such as IQ, leisure activities, occupational and educational attainment [11], early life experiences [12] and socioeconomic status (SES) [13]. The Cognitive Reserve Index Questionnaire (CRIq) [11] is a multi-dimensional tool that measures cognitive reserve by calculating scores based on an individual’s years of education, working time, and frequent leisure activities. It has been widely translated [14] and validated in dementia populations [15], but has not been validated for GBM.

Cognitive deficits in GBM patients are heterogeneous, with studies showing that multiple cognitive domains are affected, including executive function, attention, and memory. The reason for this heterogeneity is unclear. It is unknown whether tumour and treatment effects, such as surgery, can be mitigated by protective factors. Despite the well-documented cognitive deficits experienced by GBM patients [16], the role of cognitive reserve remains largely unexplored. Prior studies have focused on language [17] or preoperative cognition [18]. To our knowledge, no research has evaluated multidimensional cognitive reserve, measured with the CRIq, in relation to overall cognitive outcomes across the pre- and postoperative period. It is important to understand which patients are vulnerable to cognitive deficits to stratify patients for different treatments, such as aggressive surgical resections, to balance improving survival with risk to cognition.

This study aimed to investigate the impact of cognitive reserve, as measured by the CRIq, on cognitive outcomes in GBM. We further tested whether CRIq-derived education, occupational, and leisure activity proxies predict cognition via mediation analyses. We hypothesise that cognitive reserve is associated with preoperative cognition in GBM patients. Exploring the utility of the CRIq in GBM could provide a clinically useful tool for predicting cognitive impairment and guiding personalised care.

## Methods

### Participants

This study was conducted as part of the Surgically Induced Neurological Deficits in Glioblastomas (SIND) project at one University Hospital (REC: 19/WM/0152). 43 adults with GBM or gliosarcoma were recruited and provided signed consent for all study interventions. All patients had evidence of GBM on MRI, were pathologically confirmed with IDH-wildtype status, and were felt eligible for surgery with a WHO Performance Status of 0–2. Patients were neuropsychologically screened multiple times, preoperatively and postoperatively. Four patients experienced postoperative complications. Two of these patients were excluded from postoperative analyses due to attrition. Consequently, only two patients with complications were included in the postoperative analyses. To account for the potential impact of these complications on functional status, postoperative WHO Performance Status was included as a covariate in all postoperative regression analyses.

### Materials

#### Neuropsychological screening tool

Cognitive function was screened using the Oxford Cognitive Screen (OCS) and the Cambridge Attention, Memory and Perception Screen (OCS-BRIDGE) [19]. The OCS-BRIDGE screen assesses multiple cognitive domains (language, executive function, praxis, visual acuity, memory, emotion recognition and attention) via touchscreen in ~ 25 min. The OCS has been validated for use in stroke, with high test-retest reliability even across parallel versions, and is acceptable for use in glioma patients [20, 21]. Additionally, compared to traditional methods, the OCS-BRIDGE can account for aphasia and neglect and captures reaction times, which enhances its sensitivity in non-verbal functioning [20].

Patients were assessed at T0 - baseline (< 1–2 weeks before surgery), T1 – early postoperative (72 h post-surgery) and T2 – delayed postoperative (6–8 weeks post-surgery). The 72-hour timeframe was selected to capture an acute postoperative baseline, and the 6–8-week assessment was conducted to capture delayed postoperative performance before the confounding effects of adjuvant therapy initiation. To mitigate practice effects, parallel versions of the test were administered at alternating time points.

#### Cognitive reserve proxies

Cognitive reserve was assessed using the CRIq [11], which has not been previously validated for use in HGG patients; its use here is exploratory. The CRIq captures information on years of education and vocational training, years of work, and frequency of cognitively stimulating leisure activities. Four scores were generated using the calculation method described by Nucci et al. (2012): CRI-Education (CR-E), CRI-Working Activity (CR-W), CRI-Leisure Time (CR-L) and CR-I (overall scores) [11]. A composite CR score for leisure time and working activity (CR-WL) was calculated as the mean of the CR-L and CR-W scores. This approach mirrors the method used by Nucci et al. (2012), which involved averaging the three subscores and standardising the result to a mean of 100 and a standard deviation of 15.

### Statistical analysis

All statistical analyses were conducted using R (version 4.4.3).

#### Composite scores

Participants’ scores on the OCS-BRIDGE screening tests were compared to a normative sample to create z scores. Deficits in particular tests were determined by a z score of less than − 1.645, representing the 5% impairment cut-off recommended in the OCS-BRIDGE manual. Each test corresponded to a specific domain designated in the OCS-BRIDGE battery, which is included in the supplementary material alongside more detailed information about each test. Domain deficit percentages were then calculated by dividing the number of tests failed in that domain by the total number of tests in that domain. A global deficit percentage was calculated by dividing the total number of tests failed by the total number of tests taken. Median domain scores were also calculated by calculating the median of the z scores across the tests corresponding to a domain.

#### Linear regressions

Linear regressions were run to assess the direct relationship between overall cognitive reserve (CR-I) and pre- and postoperative cognitive performance (the global deficit percentage, domain deficit percentages and median domain z-scores). Domains for regressions were selected based on previous literature linking them with cognitive reserve (Executive Function, Attention, Memory, Language and Recognition Memory [22, 23]). All regressions visually met the assumptions of linearity, homoscedasticity and normality of residuals. Each linear regression controlled for sex, age, lesion volume, tumour side, and WHO performance status binarised (WHO Status 0 versus WHO Status 1 and 2). To prevent overfitting of the model given the sample size, lesion volume was the only proxy for tumour burden. Fluid-Attenuated Inversion Recovery (FLAIR) volume was excluded as preliminary analyses showed it was not significantly associated with cognitive performance. False discovery rate (FDR) was applied to account for multiple testing.

#### Mediation analyses

Mediation analyses were conducted with the mediation package in R using 5000 bootstrap resamples (seed = 123) [24]. Analyses were only conducted on variables that were identified as being significantly related to CR-I in the linear regressions. The model tested the a-path (effect of CR-E on CR-WL), and the b-path and c’-path (effects of CR-E and CR-WL on the domains). The mediator model (a-path) controlled for age and sex, while the outcome models (b and c’ paths) additionally controlled for WHO performance status binarised, lesion volume and tumour side. All models met the assumptions of linearity, normality of residuals, and no multicollinearity (VIF < 2). The indirect effect was estimated using 5000 resamples.

## Results

### Demographic and clinical characteristics

**Fig. 1:**
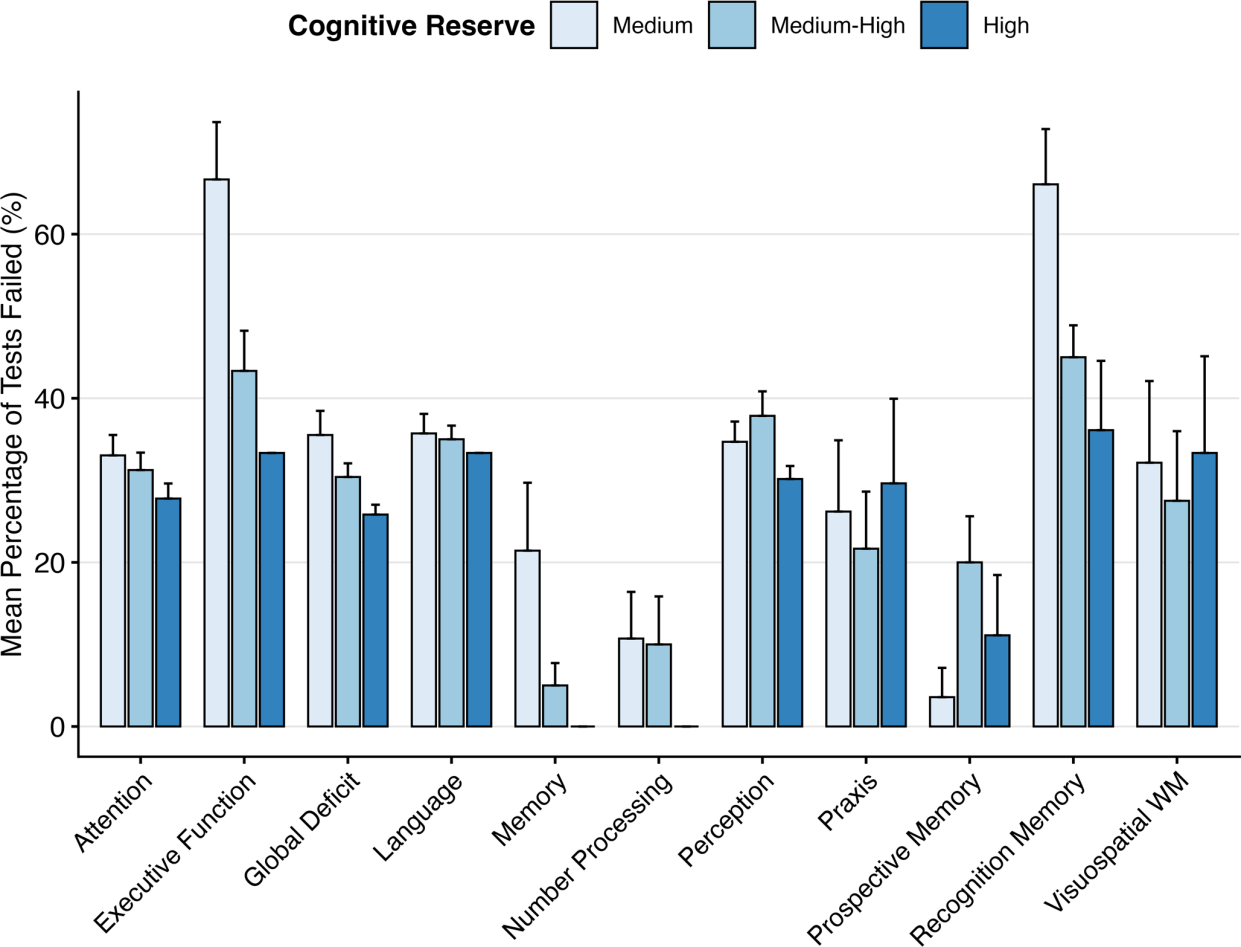
Bar chart, stratified by cognitive reserve score, to illustrate preoperative cognitive impairment across domains. Profile of preoperative cognitive impairment across domains stratified by Cognitive Reserve Index (CR-I) group. The height of each bar represents the mean percentage of tests failed within that cognitive domain, with higher values indicating greater deficit. Error bars represent the standard error of the mean (SEM). Patients are grouped by cognitive reserve level, with lighter shades indicating lower reserve (Medium; *N* = 13), (Medium-High; *N* = 20) and darker shades indicating higher reserve (High; *N* = 9). (Visuospatial WM = Visuospatial Working Memory)

The demographic and clinical characteristics of the 43 participants are summarised in Table 1. The cohort consisted predominantly of patients with IDH-wildtype GBM (93%) and Gliosarcoma (7%). The mean age was 62 years, and most patients presented with a WHO performance status of 0 or 1. Preoperative cognitive impairment profiles, stratified by CR-I group, are displayed in Fig. 1. 

**Table 1:** Summary of Demographic and Clinical Characteristics of the Patient Cohort (*N* = 43)

Characteristic	N = 43^1^
Sex	
Male	29 (67%)
Female	14 (33%)
Tumour Side	
Right	22 (51%)
Left	21 (49%)
WHO Performance Status	
0	21 (49%)
1	20 (47%)
2	2 (4.7%)
Pathology	
GBM	40 (93%)
Gliosarcoma	3 (7.0%)
Tumour Lobe	
Frontal	20 (47%)
Temporal	13 (30%)
Parietal	8 (19%)
Occipital	2 (4.7%)
Complication	
None	39 (91%)
Yes	4 (9.4%)
IDH Status	
Wildtype	43 (100%)
MGMT Status	
Unmethylated	22 (51%)
Methylated	21 (49%)
Age (years)	62 (10)
Lesion Volume (cm³)	34 (20)
FLAIR Volume (cm³)	26 (22)
Cognitive Reserve (CR-I)	119 (13)
CR-Education (CR-E)	119 (13)
CR-Work (CR-W)	109 (14)
CR-Leisure (CR-L)	115 (18)

^1^n (%); Mean (SD)

### The relationship between cognitive reserve and cognition

First, we assessed the direct relationship between overall cognitive reserve (CR-I) and the cognitive performance scores preoperatively. Linear regression analyses revealed that higher overall cognitive reserve was significantly associated with better preoperative performance across multiple domains. Specifically, higher CR-I predicted lower deficit percentages in Global cognition (*p* = .014), Executive Function (*p* = .002), Memory (*p* = .046), and Recognition Memory (*p* = .011) after correcting for multiple comparisons (FDR). Similarly, analysis of median z-scores confirmed that higher reserve was associated with significantly better scores in Executive Function (*p* = .008) and Recognition Memory (*p* = .020), after correcting for multiple comparisons (FDR).

**Fig. 2:**
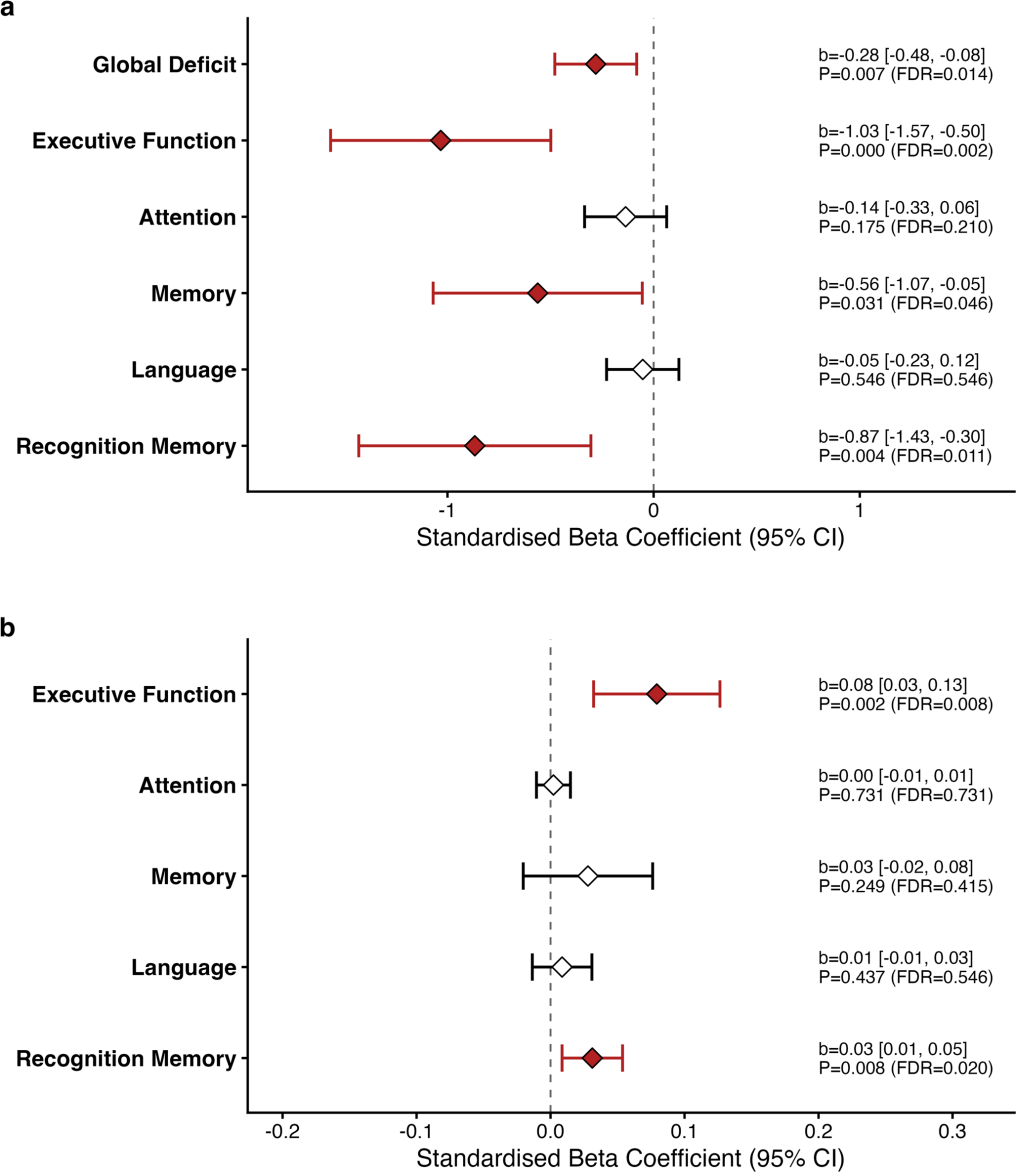
Association between cognitive reserve and preoperative cognitive performance. Forest plots showing the association between Cognitive Reserve Index (CR-I) and preoperative cognitive performance. a) Association with deficit percentages (negative estimates indicate higher reserve is linked to less impairment. b) Association with median Z-scores (positive estimates indicate higher reserve is linked to better performance). b = unstandardized beta coefficient; CI = confidence interval. Significance (FDR *p* < .05) is indicated by red diamonds

No significant association was found for the Attention or Language domains (both deficit percentages and median scores). These findings are illustrated in Fig. 2, and the full regression results are presented in Table 2.

### The relationship between postoperative cognition and cognitive reserve

Linear regressions revealed a significant relationship between median Recognition Memory score (*p* = .010, FDR *p* = .049) at the 72-hour follow-up. However, no other postoperative cognitive domains were associated with cognitive reserve. The full regression results are displayed in Table 2 below.

**Table 2:** Association between overall cognitive reserve and cognitive performance

		Beta (95% CI)	*P*-value	FDR *P*-value
Deficit Percentages				
Global	**T0**	**-0.28 [-0.48**,** -0.08]**	**0.007**	**0.014**
	T1	-0.27 [-0.54, -0.01]	0.046	0.202
	T2	-0.29 [-0.74, 0.16]	0.191	0.286
Executive Function	**T0**	**-1.03 [-1.57**,** -0.50]**	**< 0.001**	.**002**
	T1	-0.27 [-0.91, 0.38]	0.405	0.441
	T2	-0.56 [-1.36, 0.23]	0.157	0.286
Attention	T0	-0.14 [-0.33, 0.06]	0.175	0.210
	T1	-0.10 [-0.37, 0.17]	0.441	0.441
	T2	0.02 [-0.31, 0.34]	0.911	0.911
Memory	**T0**	**-0.56 [-1.07**,** -0.05]**	**0.031**	.**046**
	T1	-0.61 [-1.35, 0.13]	0.101	0.202
	T2	-0.66 [-1.67, 0.34]	0.184	0.286
Language	T0	-0.05 [-0.23, 0.12]	0.546	0.546
	T1	-0.23 [-0.62, 0.16]	0.244	0.366
	T2	-0.10 [-0.43, 0.22]	0.518	0.621
Recognition Memory	**T0**	**-0.87 [-1.43**,** -0.30]**	**0.004**	**0.011**
	T1	-0.23 [-0.62, 0.16]	0.244	0.366
	T2	-0.62 [-1.36, 0.13]	0.099	0.286
Median Scores				
Executive Function	**T0**	**0.08 [0.03**,** 0.13]**	**0.002**	.**008**
	T1	0.06 [0.01, 0.12]	0.032	0.079
	T2	0.07 [-0.01, 0.14]	0.087	0.217
Attention	T0	0.00 [-0.01, 0.01]	0.731	0.731
	T1	0.01 [-0.01, 0.02]	0.542	0.542
	T2	-0.00 [-0.01, 0.01]	0.941	0.941
Memory	T0	0.03 [-0.02, 0.08]	0.249	0.415
	T1	0.04 [-0.03, 0.12]	0.223	0.304
	T2	0.04 [-0.06, 0.13]	0.402	0.647
Language	T0	0.01 [-0.01, 0.03]	0.437	0.546
	T1	0.07 [-0.05, 0.18]	0.243	0.304
	T2	0.01 [-0.02, 0.03]	0.518	0.647
Recognition Memory	**T0**	**0.03 [0.01**,** 0.05]**	**0.008**	.**020**
	**T1**	**0.02 [0.01**,** 0.04]**	**0.010**	**0.049**
	T2	0.03 [-0.00, 0.05]	0.061	0.217

b = unstandardised beta coefficient. All models accounted for age, sex, lesion volume, tumour side and WHO Performance Status Binarised. Significance (in bold) is determined by FDR p-value < 0.05

### The associations between education and lifestyle activities and cognition

Next, we investigated the mechanism behind these findings by testing a model where the effect of a patient’s education (CR-E) on their cognitive performance was mediated by their work and leisure activity levels (CR-WL). Mediation was conducted only on domains that were significantly related to cognitive reserve in the linear regression.

Preoperatively, we observed significant total and direct effects of education on Executive Function (both deficit percentage and median), but the indirect effect via work and leisure activities was not significant.

At 72 h postoperatively, we found a significant indirect effect for Recognition Memory median scores (*b* = 0.007, *p* < .05). This indicates that for recognition memory, the protective benefit of education was significantly mediated by work and leisure activities in the immediate postoperative period.

Table 3 displays the results for the mediation models where a significant relationship was established in the primary regression analysis.

**Table 3:** Contribution of Education and Lifestyle Activities to Cognitive Performance Before and After Surgery

Cognitive Score	Time Point	Indirect Effect		Direct Effect		Total Effect	
		b	95% CI	b	95% CI	b	95% CI
Global Deficit	T0	-0.060	[-0.166, 0.016]	-0.173	[-0.425, 0.057]	-0.233	[-0.467, -0.028]
Executive Function Deficit	T0	-0.149	[-0.485, 0.039]	-0.874**	[-1.452, -0.324]	-1.023***	[-1.560, -0.502]
Memory Deficit	T0	-0.173	[-0.525, 0.037]	-0.197	[-0.857, 0.415]	-0.370	[-0.967, 0.142]
Recognition Memory Deficit	T0	-0.254	[-0.666, 0.009]	-0.328	[-0.854, 0.260]	-0.582	[-1.183, 0.008]
Executive Function Median	T0	0.009	[-0.006, 0.032]	0.076***	[0.029, 0.126]	0.085***	[0.041, 0.132]
Recognition Memory Median	T0	0.010	[0.000, 0.026]	0.010	[-0.014, 0.031]	0.020	[-0.005, 0.045]
	T1	0.007*	[0.001, 0.016]	0.006	[-0.011, 0.023]	0.013	[-0.004, 0.030]

(* = *p* < .05, ** = *p* < .01, *** = *p* < .001)

## Discussion

Research investigating the relationship between cognitive reserve and cognitive function in GBM is scarce. While cognitive reserve is a well-established protective factor in neurodegeneration [9], no prior studies have assessed whether multidimensional reserve proxies, such as those captured by the CRIq, predict multiple cognitive domains pre- and postoperatively. This study is, to our knowledge, the first to address this gap.

### Interpretation of findings

Our results indicate that higher cognitive reserve is significantly associated with better preoperative performance across global cognition, executive function, memory and recognition memory. This supports the theory that the reserve provides a “buffer” against the damage caused by the tumour, allowing patients to maintain cognitive function.

However, the mechanism of this protection appears to vary by domain. For executive function, which was most strongly associated with cognitive reserve, we found a significant direct effect of education, but the mediation pathway of working activity and leisure time was not significant. This suggests that, for executive function, educational attainment may be the primary driver of the buffer, potentially establishing resilience earlier in life and reducing reliance on later lifestyle engagement. Yet it must be considered that the relationship between executive function and education may be bidirectional: greater executive function could facilitate a person gaining more years of education and thus increase their cognitive reserve scores.

In contrast, our postoperative findings highlight a different pattern. While most of the protective impacts of cognitive reserve disappear after surgery, recognition memory at 72 h showed a significant relationship with cognitive reserve. Specifically, mediation analyses revealed a significant indirect effect through work and leisure activity for this domain. This implies that, unlike executive function, the ability to retain recognition memory during the acute post-surgery period may be supported by “active” reserve components (sustained engagement in cognitively stimulating activities) rather than education alone.

Executive function and recognition memory emerged as the only domains consistently linked to cognitive reserve, suggesting they are particularly sensitive to enriching lifestyle activities and education. Our findings align with research on dementia and neurodegenerative disorders, where higher cognitive reserve has been associated with preserved executive function [25]. However, except for recognition memory at T1, the absence of effects of cognitive reserve postoperatively suggests that surgical disruption (including resection-related changes and perioperative inflammation) may temporarily overwhelm cognitive reserve’s protective influence. Another possibility is that cognitive reserve’s benefits may only emerge later in recovery, beyond the 6–8-week postoperative period examined in this study.

### Clinical implications

This study also extends previous HGG research [17, 18], demonstrating the specific value of using a multidimensional cognitive reserve proxy. There was a dissociation observed between education being associated with preoperative executive functioning, while continued leisure and working activity (CR-WL) mediated acute postoperative recognition memory. This highlights that broad and single-factor proxies (like years of education alone) may obscure domain-specific resilience mechanisms.

These findings suggest a two-tiered approach to clinical assessment. First, since executive function was associated with years of education, clinicians could utilise educational history as a rapid risk-stratification tool. Patients with lower formal education could be highlighted as being at higher risk for executive function deficits preoperatively. Second, the finding that “active” reserve may play a role in postoperative recognition memory identifies a potential target for intervention. Unlike education, which often remains fixed in adulthood, continued years of leisure and work-related engagement are often modifiable. This supports encouraging sustained cognitive engagement prior to surgery, as it may be able to provide some resilience against the “shock” of surgical resection.

### Theoretical implications

Furthermore, these findings have theoretical implications. They highlight that the impact of cognitive reserve on cognitive function depends on the domain and context. Relying solely on education as a cognitive reserve proxy may underestimate an individual’s cognitive resilience, particularly regarding recognition memory preservation. Our results also suggest that surgical planning should not be drastically modified based on cognitive reserve, as its protective effects do not consistently extend to postoperative outcomes in the weeks after resection.

### Limitations

Several limitations should be considered. The sample size (*n* = 43) is modest, limiting statistical power. Additionally, the CRIq classified all participants as having medium or higher cognitive reserve, with no individuals in the low or medium-low categories. This may reflect inherent scoring biases: even minimal engagement in household or social activities can inflate cognitive reserve estimates. This distribution may also reflect selection biases in our recruitment region or criteria (e.g., WHO performance status), potentially masking protective effects in individuals with lower reserve. Consequently, the lack of participants with low cognitive reserve in this study may lead to an underestimation of the protective power of cognitive reserve.

There are additional limitations that need to be considered when applying the CRIq to GBM patients, as it has not been previously validated in this population. Specific challenges include retrospective reporting (as patients reflect on activity levels post-diagnosis) and the potential confounding influence of premorbid cognitive decline caused by the tumour before study entry.

Follow-up was limited to 6–8 weeks postoperatively, leaving the longer-term impacts of cognitive reserve on cognitive recovery unknown. However, the difficulty of studying longer than this is that radiotherapy and chemotherapy may also have an impact on patients. Furthermore, mediation analyses are correlational, meaning no causality can be inferred. Higher executive function or recognition memory may enable greater engagement in education, working or leisure activities, rather than the reverse. Finally, this study used a brief cognitive screen (OCS-BRIDGE) rather than a comprehensive neuropsychological battery, which may limit sensitivity to subtle cognitive changes.

## Conclusion

The present study provides exploratory support for the use of CRIq. Future research should focus on developing refined scoring systems to better capture the full spectrum of cognitive reserve. A crucial next step is to directly compare the predictive value of various reserve proxies, such as cognitive tests themselves, in the HGG population. Such work would clarify the distinct impacts of single measures (e.g., education alone) versus composite indices, guiding the development of a more robust, population-specific measure of cognitive reserve. Longitudinal research is needed to determine whether cognitive reserve’s protective effects re-emerge during long-term postoperative recovery and to assess whether targeted interventions can enhance cognitive reserve and mitigate cognitive decline in HGG patients. 

## Data Availability

The data that support the findings of this study are available on request from the corresponding author, SR. The data are not publicly available due to their containing information that could compromise the privacy of research participants.
